# Fiber-based *in vivo* imaging: unveiling avenues for exploring mechanisms of synaptic plasticity and neuronal adaptations underlying behavior

**DOI:** 10.1117/1.NPh.11.S1.S11507

**Published:** 2024-02-22

**Authors:** Anna Karpova, Ahmed A. A. Aly, Endre Levente Marosi, Sanja Mikulovic

**Affiliations:** a Leibniz Institute for Neurobiology, RG Neuroplasticity, Magdeburg, Germany; b Otto von Guericke University, Center for Behavioral Brain Sciences, Magdeburg, Germany; c Leibniz Institute for Neurobiology, RG Cognition and Emotion, Magdeburg, Germany; d German Centre for Mental Health (DZPG), Magdeburg, Germany

**Keywords:** fiber-based endo-microscopy, synaptic neurotransmission, neuronal connectivity, structural plasticity, membrane trafficking

## Abstract

In recent decades, various subfields within neuroscience, spanning molecular, cellular, and systemic dimensions, have significantly advanced our understanding of the elaborate molecular and cellular mechanisms that underpin learning, memory, and adaptive behaviors. There have been notable advancements in imaging techniques, particularly in reaching superficial brain structures. This progress has led to their widespread adoption in numerous laboratories. However, essential physiological and cognitive processes, including sensory integration, emotional modulation of motivated behavior, motor regulation, learning, and memory consolidation, are intricately encoded within deeper brain structures. Hence, visualization techniques such as calcium imaging using miniscopes have gained popularity for studying brain activity in unrestrained animals. Despite its utility, miniscope technology is associated with substantial brain tissue damage caused by gradient refractive index lens implantation. Furthermore, its imaging capabilities are primarily confined to the neuronal somata level, thus constraining a comprehensive exploration of subcellular processes underlying adaptive behaviors. Consequently, the trajectory of neuroscience’s future hinges on the development of minimally invasive optical fiber-based endo-microscopes optimized for cellular, subcellular, and molecular imaging within the intricate depths of the brain. In pursuit of this goal, select research groups have invested significant efforts in advancing this technology. In this review, we present a perspective on the potential impact of this innovation on various aspects of neuroscience, enabling the functional exploration of *in vivo* cellular and subcellular processes that underlie synaptic plasticity and the neuronal adaptations that govern behavior.

## 1 Evolution of *In Vivo* Deep Brain Imaging Technologies

**Fig. 1:**
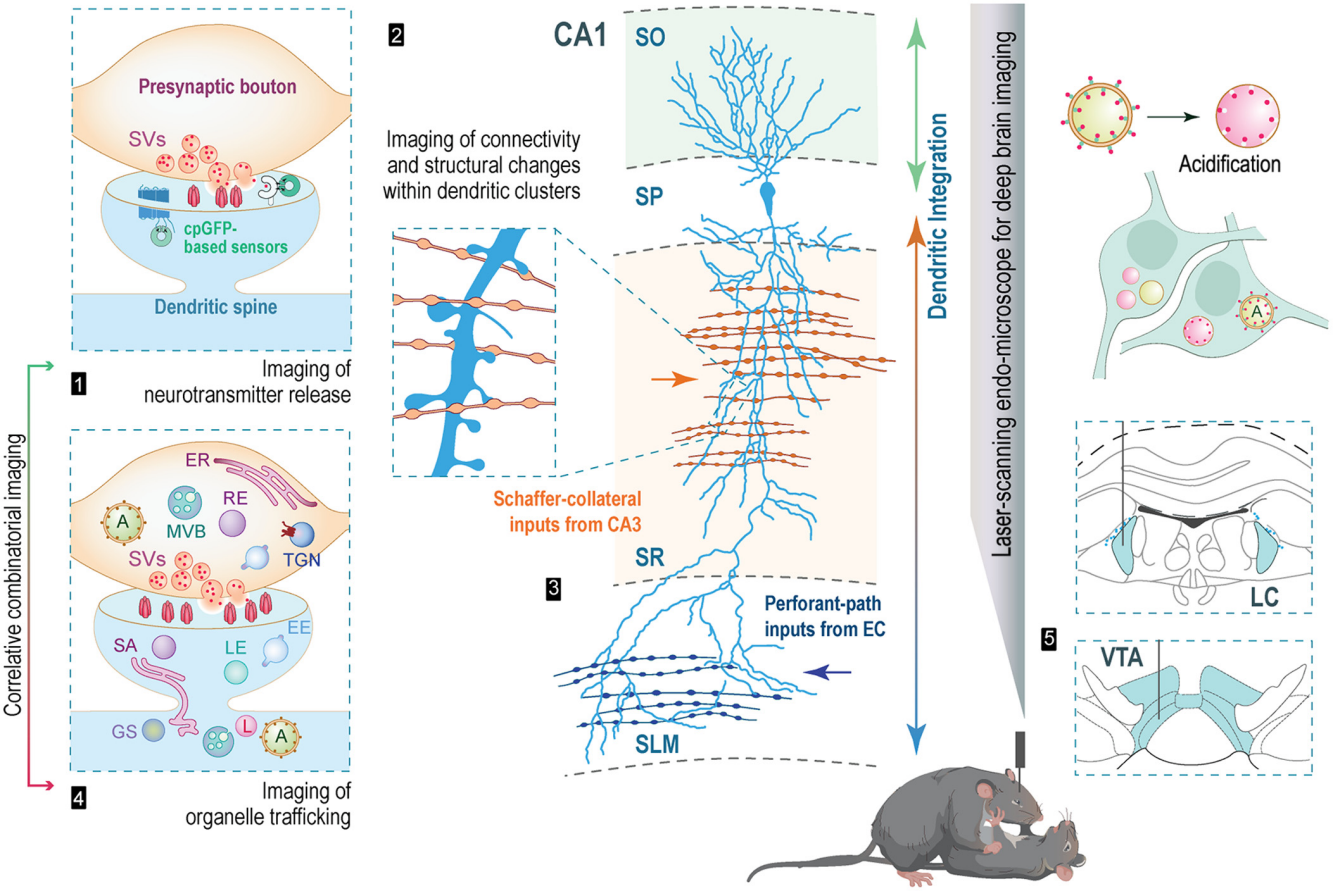
Perspectives on *in vivo* fiber-based imaging: synaptic neurotransmission, neuronal connectivity, structural plasticity, and membrane dynamics in deep brain structures in awake animals. (1) Schematic overview of imaging of synaptic neurotransmission using cpGFP-based reporters designed to detect various neurotransmitters such as glutamate, acetylcholine, GABA, norepinephrine, dopamine, etc. SVs, synaptic vesicles. (2) Structural imaging of dendritic compartmentalization down to the resolution of a single dendritic spines, as well as imaging of neuronal connectivity while animals are actively engaged in learning tasks or social behavior. (3) A schematic drawing of a CA1 pyramidal neuron, depicting distinct dendritic domains capable of receiving unique synaptic inputs. Proximal-to-distal arrows depict dendritic integration. CA1, cornu ammonis 1 subfield of the hippocampus; SO, stratum oriens; SP, stratum pyramidale; SR, stratum radiatum; SLM, stratum lacunosum moleculare; EC, entorhinal cortex. (4) Imaging of membranous organelle trafficking *in vivo* in correlation with neuronal activity in awake animals. A, autophagosome; ER, endoplasmic reticulum; MVB, multivesicular bodies; RE, recycling endosome; TGN, trans-Golgi network; SA, spine apparatus; LE, late endosome; GS, Golgi satellites; L, lysosome; EE, early endosome; LE, late endosome. (5) The schematic depicts organelle labeling with an acidification probe within neuronal somata of the VTA and LC for *in vivo* imaging using dual-color ultra-thin fiber-based imaging technology. (Images of animals were obtained from www.biorender.com.)

The complexity of the brain demands comprehensive methodologies for exploring the interconnected brain regions and understanding how neuronal communication shapes behavior. Highly polarized neurons in the brain feature a numerous *en passant* presynaptic boutons [Fig. 1(1)], which are crucial for neurotransmitters and neuromodulators release.^1^ In addition, they possess postsynaptic sites that extend along their dendrites, often associated with compartmentalized dendritic spines or positioned within the dendritic shaft itself.^2^^,^^3^ These intricate structural elements collectively enable an adaptable mode of cell-to-cell communication known as synaptic transmission.^4^ The consolidation of diverse forms of memories and the neuronal adaptations underlying behavior are orchestrated by the coordinated engagement of pre- and postsynaptic mechanisms within specific brain circuits.^5^^–^^11^

Two-photon (2P) microscopy has emerged as a promising tool to monitor neuronal activity in behaving animals.^12^ This technique has undergone continuous refinements, driven by both software-^13^ and hardware-based advancements, including adaptive optical correction of aberrations,^14^^,^^15^ three-photon approaches,^16^^–^^18^ and the design of red-shifted indicators, which reduce the scattering of the emitted light, further improving imaging depth.^19^^,^^20^ Despite the substantial progress achieved in the past two decades, extending imaging depth remains a challenge. This was initially achieved through the implementation of rod-shaped gradient refractive index (GRIN) lenses, typically ranging in diameter from 500 to 1000  μm. These innovations paved the way for the development of miniscopes, enabling imaging in unrestrained animals.^21^^–^^26^ While GRIN lens implantation is commonly described as a minimally invasive imaging technique, it is essential to recognize that it can still cause substantial tissue damage. Consequently, this damage can lead to significant necrosis and gliosis that may persist for weeks after surgery.^27^ Furthermore, the postsurgical effects have the potential to induce artificial dendritic arbor reorganization.^28^

In the field of neurophotonics, recent breakthroughs with the implementation of multimode fiber-based imaging techniques,^29^ allowing imaging through ultra-thin fibers, have yielded significant advancements for the *in vivo* brain imaging.^30^ Subsequently, these led to the creation of a remarkable 110  μm thin laser-scanning endo-microscope.^31^^,^^32^ This imaging technology is characterized by the minimal tissue damage, sub-1  μm lateral resolution, three-dimensional random-access capabilities, and multiwavelength detection. Furthermore, the fiber small size allows precise positioning within the region of interest, enabling the assessment of neural activity in various dendritic branches and axonal subregions in behaving animals—a distinct advantage not present in GRIN lens technology.

## 2 Combinatorial Imaging of Synaptic Neurotransmission, Neuronal Connectivity, and Structural Changes Underlying Plasticity

Understanding the correlation between adaptive behavior and the interplay of presynaptic release, neuronal connectivity, and structural changes in dendritic branches up to the resolution of single dendritic spines and synaptic boutons in the brains of actively engaged animals is of crucial importance. Recent collaborative efforts among genetic engineers and molecular biologists have provided a large repertoire of advanced molecular probes enabling the assessment of neurotransmission in correlation with structural modifications in synaptic compartments [Fig. 1(1)]. Among these probes, there are several generations of single-wavelength reporters designed to sense glutamate, γ-aminobutyric acid (GABA), norepinephrine, dopamine, etc. The first group includes circularly permuted green fluorescent protein (cpGFP)-based iGluSnFR,^33^^–^^35^ a membrane-tethered version based on cp superfolder GFP, sf-iGluSnFR, with specific point mutations enabling high spatial and temporal precision in glutamate detection,^36^^,^^37^ an improved version of sf-iGluSnFR featuring enhanced trafficking to the plasma membrane through the addition of Golgi export- and an endoplasmic reticulum (ER) exit motif sequences,^38^ and a red glutamate sensor based on cp mApple, R-iGluSnFR1.^39^ The imaging of inhibitory neurotransmission can be effectively accomplished by employing either iGABASnFR or iGABASnFR.mRubi3.^40^ Several generations of cpGFP-based norepinephrine sensors, with the latest developments referred to as GRAB-NE3.1 and GRAB-NE2h,^41^^,^^42^ are complemented by a range of dopamine sensors extending the dLight1 family,^43^^–^^47^ allowing imaging of modulatory inputs. An approach, utilizing fluorescent protein reconstitution at synaptic sites with probes such as mGRASP^48^^–^^50^ dual-eGRASP,^51^ can effectively label synaptic connections within neuronal circuits. Furthermore, the recent advancement in the SynapShot probe, utilizing dimerization-dependent fluorescent proteins, enables the real-time observation of reversible and bidirectional alterations in synaptic contacts during physiological stimulation and enables real-time monitoring of structural changes in synaptic contacts in the brain of behaving mice.^52^ In addition, Split Protein HEmispheres for REconstitution (SPHERE) technology for both iGluSnFR and iGABASnFR probes has been recently established enabling separate expression in pre- and postsynaptic neurons parts of splitted iGluSnFR and iGABASnFR for the functional detection of neurotransmitters at the contact sites.^53^ The latter approach provides the opportunity to simultaneously assess functional synaptic neurotransmission and neuronal connectivity for evaluating circuit-related behaviors. In combination with ultra-thin fiber imaging technology, these approaches would enable *in vivo* imaging of functional neuronal connectivity within the circuit of interest.

While the structural plasticity of dendritic spines, including spine motility, stability, experience-dependent growth, as well as axon branching, synaptic bouton dynamics, and synaptic structure turnover within cortical and dorsal hippocampal areas has been extensively studied,^54^^–^^60^ its longitudinal examination in deep brain regions of behaving animals remains relatively unexplored. Synaptic distribution within a neuron’s dendritic arbor is organized into functional clusters and critical for the segregation of synaptic inputs, the phenomenon, known as “clustered synaptic plasticity.”^61^^–^^65^ In conjunction with clustered synaptic plasticity, dendritic compartmentalization entails the division of a neuron’s dendritic tree into discrete segments, each with unique functional properties [Figs. 1(2) and 1(3)].^66^^,^^67^ Furthermore, the application of ${\mathrm{Ca}}^{2+}$ imaging in deep tissue has the potential to significantly enhance our understanding on multiple aspects of ${\mathrm{Ca}}^{2+}$ signaling and ${\mathrm{Ca}}^{2+}$ homeostasis. Beyond serving as a valuable tool for providing precise spatiotemporal resolution of axonal spiking activity,^68^ imaging of ${\mathrm{Ca}}^{2+}$ transients delves into various facets of ${\mathrm{Ca}}^{2+}$ homeostasis, including residual ${\mathrm{Ca}}^{2+}$ concentration, clearance, decay, and summation. Although these aspects have been extensively studied *in vitro*,^69^^–^^72^ the question of the relevance of presynaptic heterogeneity in ongoing behavior remains an open inquiry. Therefore, imaging of ${\mathrm{Ca}}^{2+}$ transients within subcellular compartments, such as mossy fiber axons in the memory-related brain region, dentate gyrus, in combination with engram technologies such as mGRASP based c-Fos or Arc-dependent labeling of synaptic engrams,^48^^,^^50^^,^^51^ enables the comparison of residual $Ca^{2}$ dynamics between engram and non-engram mossy fiber boutons during memory retrieval.^73^ This comparative analysis sheds light on the mechanistic underpinnings of function-dependent processing in these synapses, where the coupling between the action potential and the calcium influx (AP-ICa coupling) is indistinguishable.^74^

Thus, although the application of ultra-thin endo-microscope technology already extends investigations into synaptic neurotransmission, neuronal connectivity, and structural changes underlying plasticity within deep brain regions, further improvement in this technique is adequate. In the future, this technology should enable *in vivo* examination of the organization and function of axonal boutons larger than 1  μm, including corticothalamic boutons^75^ and the mnemonic calyx of Held,^76^ while also allowing imaging of wider areas of dendritic branches and axonal arbors.

## 3 Exploring Membrane Trafficking *In Vivo* in Correlation with Neuronal Activity and Animal Behavior

The ultra-thin laser-scanning endo-microscope allows for subcellular membranous compartment resolution, including vesicular structures of the size of lysosomes.^31^ This offers new prospects for *in vivo* subcellular imaging, encompassing multiple membranous compartments, their functional attributes, and trafficking patterns in response to neuronal activity. A subset of membrane trafficking processes includes trafficking of organelles to specific locations within neurons. This ensures the timely delivery of essential building blocks, proteins, and protein complexes while also facilitating a delivery of post-translationally modified transmembrane proteins and receptors to the neuron’s plasma membrane through outward biosynthetic-secretory membrane trafficking.^77^^–^^83^ The trafficking of double-membrane autophagosomes^84^^,^^85^ and amphisomes,^86^^–^^89^ which subsequently fuse with lysosomes, plays a critical role in maintaining neuronal proteostasis and is integral to the degradation of unwanted cellular cargos such as misfolded proteins and even damaged organelles.^90^ While there is a continuous exchange of membranes taking place, specific resident proteins or peptides are efficiently employed to target fluorescent proteins to particular organelles and allow for their visualization in living cells [Fig. 1(4)]. Widely used example probes include the GFP-KDEL probe, utilized for ER labeling,^91^ KFERQ-sequence fused to Dendra for imaging of chaperone-mediated autophagy,^92^ YFP-ERGIC-53, a marker indicating ER-Golgi Intermediate Compartment (ERGIC),^93^^,^^94^ GM130, which labels somatic Golgi,^77^ TGN38 for Trans-Golgi-Network labeling,^83^ the pGolt-mCherry probe for Golgi satellites labeling,^81^^,^^82^^,^^95^ Rab5 for early- and Rab7 for late-endosome labeling,^96^ the autophagy-related protein (ATG) ATG8/LC3 for labeling a subset of autophagosomes,^97^ and LAMP1 and LAMP2 for lysosome labeling.^97^^–^^101^ Furthermore, alterations in organelle function can be monitored in various animal models of neurodevelopmental and neurodegenerative disorders, particularly in the deep brain regions such as ventral tegmental area (VTA) and locus coeruleus (LC), which are particularly vulnerable to neurodegeneration. For example, the tandem mRFP-GFP fusion with LC3, serving as an acidification probe, differentiates non-acidified autophagosomes labeled with the dual signal from acidified autolysosomes marked with mRFP due to their low pH environment.^102^ The combination of dual color ultra-thin fiber-based imaging technology and the use of the acidification probe may provide insightful information on alteration in neuronal proteostasis in these brain regions underlying neurodegeneration [Fig. 1(5)]. Moreover, the scientific community has access to diverse mouse models expressing specific organelle markers.^82^^,^^92^^,^^103^^,^^104^ Thus, circuit-specific organelle labeling approach, when combined with ultra-thin fiber-based imaging technology, provides a novel perspective for visualizing membrane trafficking and specific organelle functions in deep brain regions of both healthy and diseased animals.

## 4 Limitations and Future Developments

In this review, we have centered our discussion on elucidating the perspectives and emerging opportunities afforded by the development of ultra-thin fiber imaging technology. Although the challenge of achieving longitudinal imaging with the immersible endo-microscope remains unresolved, the potential to enable the continuous observation of neural connections across consecutive days, may be enabled by custom connector solutions or exploitations of implantable thin-wall guiding glass tubes,^23^ thereby providing valuable insights into the dynamics of learning or behavioral assessments.

The integration of GRIN lenses into miniscope applications has marked a significant advancement in neuroscience over the past decade, enabling the observation of brain activity in real-time during ongoing behavior. Looking ahead, we envision a synergistic relationship between these two technologies, tailored to the specific scientific inquiries. While a GRIN lens boasts a larger size and an expanded field of view, the microendoscope is constrained to ∼100  μm in diameter, making it better suited for very deep brain structures. Furthermore, the GRIN lens demonstrates compatibility with 2P excitation, facilitating exploration of deeper regions at the GRIN lens output. In contrast, the multimodal fiber endoscope is currently limited to one-photon excitation compatibility. While the microendoscope requires a digital micromirror device and calibration, making it currently more challenging to employ, ongoing developments by the growing scientific community holds promise in developing more user-friendly solutions in the near future. The potential complementarity of GRIN lenses and microendoscope technologies underscores their collective capability to address diverse scientific questions. In summary, the utilization of this imaging technology in freely moving animals introduce a transformative advancement, opening up new possibilities for the investigating cellular, subcellular, and molecular processes.

## Data Availability

Data sharing is not applicable to this article, as no new data were created or analyzed.
